# Methylcobalamin: A Potential Vitamin of Pain Killer

**DOI:** 10.1155/2013/424651

**Published:** 2013-12-26

**Authors:** Ming Zhang, Wenjuan Han, Sanjue Hu, Hui Xu

**Affiliations:** Institute of Neurosciences, The Fourth Military Medical University, Xi'an 710032, China

## Abstract

Methylcobalamin (MeCbl), the activated form of vitamin B12, has been used to treat some nutritional diseases and other diseases in clinic, such as Alzheimer's disease and rheumatoid arthritis. As an auxiliary agent, it exerts neuronal protection by promoting regeneration of injured nerves and antagonizing glutamate-induced neurotoxicity. Recently several lines of evidence demonstrated that MeCbl may have potential analgesic effects in experimental and clinical studies. For example, MeCbl alleviated pain behaviors in diabetic neuropathy, low back pain and neuralgia. MeCbl improved nerve conduction, promoted the regeneration of injured nerves, and inhibited ectopic spontaneous discharges of injured primary sensory neurons. This review aims to summarize the analgesic effect and mechanisms of MeCbl at the present.

## 1. Introduction

**Figure 1:**
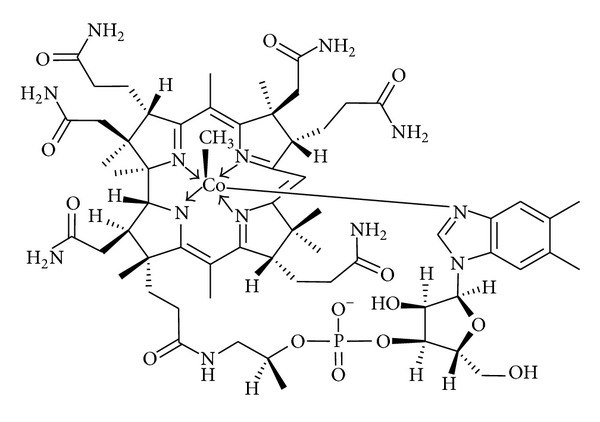
The chemical structure of MeCbl.

Vitamin B12 had been usually treated as sport nutrition, and used to keep old people from getting anemic in past years. Vitamin B12 was regarded as painkilling vitamin in some countries from 1950. Recently studies have shown that vitamin B12 played a key role in the normal functioning of the brain and nervous system and the formation of blood. Vitamin B12 is normally involved in several metabolisms such as DNA synthesis and regulation, fatty acid synthesis, and energy production. Vitamin B12 has some analogs including cyanocobalamin (CNCbl), methylcobalamin (MeCbl), hydroxocobalamin (OHCbl), and adenosylcobalamin (AdoCbl). In mammalian cells, CNCbl and OHCbl are inactive forms and AdoCbl acts as a coenzyme of methylmalonyl Co-A mutase in mitochondria. However, vitamin B12 was not used directly in human body, and it should be translated into activating forms such as MeCbl or AdoCbl. MeCbl differs from vitamin B12 in that the cyanide is replaced by a methyl group (Figure 1) [1]. It is a coenzyme of methionine synthase, which is required for the formation of methionine from homocysteine in the methylation cycle which involves methylation of DNA or proteins [2–5]. Compared with other analogs, MeCbl is the most effective one in being uptaken by subcellular organelles of neurons. Therefore, MeCbl may provide better treatments for nervous disorders through effective systemic or local delivery.

As an auxiliary agent, MeCbl has been always used to treat many diseases, such as B12 deficiency and Alzheimer's disease syndromes [6, 7]. L-methylfolate, MeCbl, and N-acetylcysteine improved memory, emotional functions, and communication with other people among Alzheimer's patients [7, 8]. MeCbl also has neuronal protection including promoting injured nerve and axonal regeneration [9, 10] and confronting against glutamate-induced neurotoxicity [9, 11]. In addition, MeCbl improved nerve conduction in either patients of diabetic neuropathy [12–14] or streptozotocin-diabetic rats [15] and experimental acrylamide neuropathy [16]. MeCbl also improved visual function [17], rheumatoid arthritis [18], Bell's palsy, and sleep-wake rhythm disorder [19, 20]. Recently, MeCbl has been demonstrated to have potential analgesic effects on neuropathic pain in experimental and clinical studies.

## 2. The Analgesic Effect of MeCbl

MeCbl is one active form of vitamin B12 which can directly participate in homocysteine metabolism. More and more researches showed that MeCbl has beneficial effects on clinical and experimental peripheral neuropathy.

### 2.1. Diabetic Peripheral Neuropathic Pain

Clinical symptoms in legs, such as paresthesia, burning pains, and spontaneous pain, were ameliorated by MeCbl [21, 22] (Table 1). The effects of single use of MeCbl or combined use with other drugs were reviewed in diabetic neuropathy pain [12, 23] (Table 1). Clinical evidence proved that MeCbl had the capacity to inhibit the neuropathic pain associated with diabetic neuropathy.

The intensity of the pain is variable and may be described as a hot, burning, cold, aching, or itching sensation with, at times, increased skin sensitivity. In clinics, it is still a challenge to treat diabetic neuropathic pain. Carbamazepine and dolantin were not able to relieve these symptoms. Similarly, therapeutic effects of aldose reductase inhibitors and nimodipine were not encouraging in clinic as much as basic studies showed. Fortunately, MeCbl may bring a glimmer of hope to treat diabetic neuropathic pain.

### 2.2. Low Back Pain

Between 70 and 80% adults have experienced low back pain at some times in their life [24]. Back pain is one of the most common health complaints. But the causes are extensive, cancer, infection, inflammatory disorders, structural disorders of the spine itself, and disk herniation, are somewhat more common, and together account for back pain. It is supposed that the MeCbl is becoming a decent choice for the therapy to the chronic low back pain. Neurogenic claudication distance was improved significantly after the application of MeCbl [25] (Table 2). However Waikakul's research demonstrated that MeCbl was not good for pain on lumbar spinal stenosis [25]. In a trial, the analgesic effect of MeCbl has been investigated in nonspecific low back pain patients with intramuscular injection [26] (Table 2). The inconsistent effect of MeCbl might be due to different causes of lumbar spinal stenosis and nonspecific low back pain. Further studies are needed to determine the effect of MeCbl on low back pain.

### 2.3. Neck Pain

Chronic neck pain is becoming a common problem in the adult population, for the prevalence of 30%–50% in 12 months [27, 28]. It was shown that spontaneous pain, allodynia, and paresthesia of patients with neck pain were improved significantly in the MeCbl group, and with the increase of treatment time of MeCbl, the analgesic effect was more obvious [29] (Table 2).

### 2.4. Neuralgia

#### 2.4.1. Subacute Herpetic Neuralgia

The treatment of MeCbl significantly reduced continuous pain, paroxysmal pain, and allodynia in the subacute herpetic neuralgia (SHN) patients [30] (Table 3). Thus, MeCbl may be an alternative candidate for treating SHN.

#### 2.4.2. Glossopharyngeal Neuralgia

Glossopharyngeal neuralgia (GPN) is a common facial neuralgia in the pain clinics. It was reported that the numerical pain scales were decreased substantially with the treatment of MeCbl combined with gabapentin and tramadol in GPN patients [31] (Table 3). And degree of interference in quality of life including mood, interpersonal relationship, and emotion was improved earlier [31].

#### 2.4.3. Trigeminal Neuralgia

The pain of trigeminal neuralgia (TN) can be described as agonizing, paroxysmal and lancinating which may be activated by small activities such as chewing, speaking, and swallowing. A clinical trial proved that the pain of TN patients was alleviated greatly in the MeCbl group, and no recurrence of TN in pain symptoms was closed to 64% [32] (Table 3).

### 2.5. Neuropathic Pain of Animal Models

**Figure 2:**
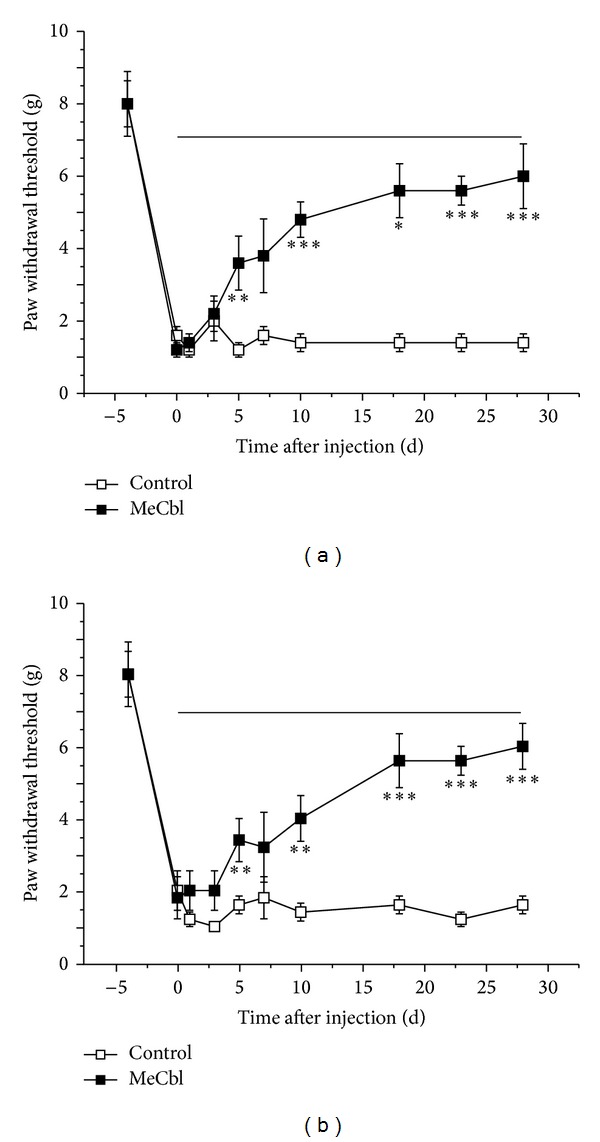
An anti-allodynic effect of MeCbl. MeCbl was successively received by intraperitoneal injections from the 3rd postoperative day (line segment). Bilateral paw withdrawal thresholds to von Frey filaments were decreased following a long-term application of MeCb1. (a) Ipsilateral side and (b) contralateral side. (*P < 0.05, **P < 0.01, ***P < 0.001, multivariate analysis of variance).

The coapplication of MeCbl and pioglitazone dramatically decreased allodynia and hyperalgesia in diabetic rats [33]. And the combined application of MeCbl and vitamin E alleviated thermal hyperalgesia in sciatic nerve crush injured rats [34]. Our recent work observed that tactile allodynia was markedly alleviated following a chronic treatment of MeCbl injection in chronic compression of dorsal root ganglion (CCD) rats (Figure 2).

## 3. Mechanisms Underlying the Analgesic of MeCbl

For many years, the B12 group of vitamins had been used to treat pain. In some countries, vitamin B12 was categorised as an analgesic drug. It was suggested that vitamin B12 may increase availability and effectiveness of noradrenaline and 5-hydroxytryptamine in the descending inhibitory nociceptive system [35]. MeCbl exerted therapeutic effects on neuropathic pain in diabetics, possibly through its neurosynthesis and neuroprotective actions [13, 36]. But the analgesic mechanisms of MeCbl remained elusive till now.

### 3.1. Improving Nerve Conduction Velocity

Previous studies showed that high doses of MeCbl improved nerve conduction in either patients with diabetic neuropathy [12–14], streptozotocin-diabetic rats [15], or experimental acrylamide neuropathy [16]. Morphological and histological evidence confirmed that a long-term administration of MeCbl promoted the synthesis and regeneration of myelin [37]. These morphological and histological recoveries of myelin may result in improving nerve conduction velocity and neuronal function in peripheral neuropathy.

### 3.2. Promoting the Regeneration of Injured Nerves

MeCbl advanced the incorporation of radioactive leucine into the protein fraction of the crushed sciatic nerve in vivo. As a result, the activity abilities of injured nerve were recovered [38]. In this study, the most terminals were degenerated in the mutant mouse, but the sprouts were more frequently observed in the MeCbl treatment group [39]. MeCbl had the ability to promote the injured nerves regeneration. In the experimental acrylamide neuropathy and sciatic nerve injury models, the number of regenerations of motor fibers showed significant increase with high-dose methylcobalamin [16]. And the combined use of L-methylfolate, MeCbl, and pyridoxal 5′-phosphate improved the calf muscle surface neural density [40].

### 3.3. Inhibiting Ectopic Spontaneous Discharge

**Figure 3:**
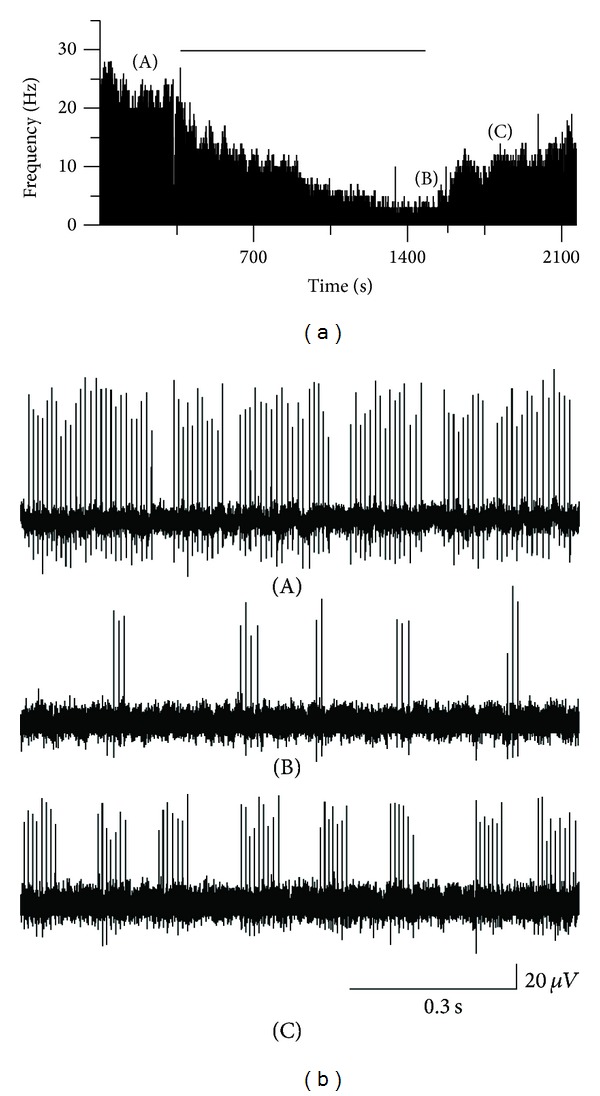
Inhibitory effect of MeCbl on ectopic spontaneous discharges of dorsal root. (a) Time histogram showing that local application of the MeCbl (300 *μ*mol/L) decreased the basal firing rate of dorsal roots. (b) Three traces in right panel show firing patterns before (A), during (B), and wash out (C) the application of MeCbl.

Ectopic spontaneous discharges are likely to initiate spontaneous pain, hyperalgesia, and allodynia [41–45]. It was reported that MeCbl suppressed the ectopic firing induced by chemical materials in the dog dorsal root [46]. Our recent work demonstrated that MeCbl markedly inhibited the ectopic spontaneous discharges of dorsal root ganglion neurons in CCD rats (Figure 3). Our results suggested that MeCbl exhibited its anti-allodynic effect by inhibiting peripheral pain signals.

## 4. Conclusions

MeCbl or its combined use with other agents has the potential analgesic effect in specific patients and animal models, for example, nonspecific low back pain; neck pain; diabetic neuropathic pain, subacute herpetic neuralgia, glossopharyngeal neuralgia, and trigeminal neuralgia. However, its mechanisms underlying the analgesic effect were poorly understood. On the basis of recent work, the possible mechanisms can be considered as follows. (1) MeCbl improved nerve conduction velocity; (2) MeCbl promoted injured nerve regeneration, recovering the neuromuscular functions in peripheral hyperalgesia and allodynia; and (3) MeCbl inhibited the ectopic spontaneous discharges from peripheral primary sensory neurons in neuropathic pain states. As a vitamin, MeCbl may be a potential candidate for treating peripheral neuropathy with good safety.

## Figures and Tables

**Table 1:** The analgesic effect of MeCbl or combined use with other drugs on patients with diabetic neuropathic pain.

Effects of MeCbl	Indices	Measures of intervention	Reference
Alleviation of neuropathic pain symptoms; improved nerve conduction velocity	Pain scale scores of patients; measure of nerve conduction velocity	Oral administration of MeCbl for 3 months	Devathasan et al. [12]

Improved nerve conduction velocity	Measure of nerve conduction velocity	Intravenous administration of MeCbl	Ishihara et al. [14]

Improved the symptoms of paresthesia, burning pains, and heaviness; no effect on nerve conduction velocity	Pain symptoms; measure of nerve conduction velocity	Repeated intrathecal injection of MeCbl at a high dose of 2.5 mg/10 mL	Ide et al. [21]

Relieved spontaneous pain by 73%	Likert-type pain intensity scale; Patients' Global Impression of Change (PGIC) scale	Intramuscular injection of MeCbl for four weeks followed by oral administration of MeCbl for additional eight weeks	Li [22]

Relieved pain and paresthesia; improved motor and sensory nerve conduction velocity	Neurolgical disability score for the grades of pain and paresthesia	Intravenous injection of MeCbl for 6 weeks	Kuwabara et al. [13]

Reduced pain scores and good tolerance	Visual analog scale and chemical safety	Oral administration of immediate-release methylcobalamin and sustained-release pregabalin for 2 weeks.	Dongre and Swami [23]

**Table 2:** The analgesic effects of MeCbl on low back pain and neck pain in clinical trials.

Effects of MeCbl	Indices	Measures of intervention	Reference
Relieved spontaneous pain, allodynia, and paresthesia.	Pain symptoms of patients with neck pain	Oral administration of MeCbl for 4 weeks	Hanai et al. [29]

Amelioration of neurogenic claudication distance; no effect on pain improvement and neurological signs	Pain symptoms; measure the neurogenic claudication distance of patients with degenerative lumbar spinal stenosis	Oral administration of MeCbl as an adjuvant medication for 6 months	W. Waikakul and S. Waikakul [25]

Reduced pain	Oswestry disability index questionnaire (ODI) and visual analogue scale (VAS) pain score of patients with nonspecific low back pain	Intramuscular injection of MeCbl for 2 weeks	Chiu et al. [26]

**Table 3:** The analgesic effect of MeCbl or combined with other agents on neuralgia.

Effects of MeCbl	Indices	Measures of intervention	Reference
Reduced or eliminated pain symptoms	Pain scales in patients with trigeminal neuralgia	Intravenous injection of MeCbl at a single dose of 0.5 mg	Teramoto [32]

Relieved overall pain, continuous spontaneous pain, paroxysmal pain, and allodynia	Likert-type pain intensity scale; Patients' Global Impression of Change (PGIC) scale	Local subcutaneous injection of MeCbl for 4 weeks	Xu et al. [30]

Lowered pain intensities; improved pain relief; reduced pain interference with quality of life	Numerical pain scale and brief pain inventory of glossopharyngeal neuralgia	Oral administration of gabapentin, tramadol, and MeCbl (0.5 mg)	Singh et al. [31]
